# Connectome-Based Neurosurgery in Primary Intra-Axial Neoplasms: Beyond the Traditional Modular Conception of Brain Architecture for the Preservation of Major Neurological Domains and Higher-Order Cognitive Functions

**DOI:** 10.3390/life14010136

**Published:** 2024-01-18

**Authors:** Marcello Magnani, Arianna Rustici, Matteo Zoli, Constantin Tuleasca, Bipin Chaurasia, Enrico Franceschi, Caterina Tonon, Raffaele Lodi, Alfredo Conti

**Affiliations:** 1 IRCCS Istituto Delle Scienze Neurologiche di Bologna, UOC Neurochirurgia, 40123 Bologna, Italy; marcello.magnani3@studio.unibo.it; 2 Dipartimento di Scienze Biomediche e Neuromotorie (DIBINEM), Università di Bologna, 40123 Bologna, Italy; arianna.rustici2@unibo.it (A.R.); matteo.zoli4@unibo.it (M.Z.); caterina.tonon@unibo.it (C.T.); raffaele.lodi@unibo.it (R.L.); 3 IRCCS Istituto Delle Scienze Neurologiche di Bologna, UOSI Neuroradiologia, Ospedale Maggiore, 40138 Bologna, Italy; 4 Programma Neurochirurgia Ipofisi—Pituitary Unit, IRCCS Istituto Delle Scienze Neurologiche di Bologna, 40121 Bologna, Italy; 5 Department of Neurosurgery, University Hospital of Lausanne and Faculty of Biology and Medicine, University of Lausanne, 1015 Lausanne, Switzerland; constantin.tuleasca@chuv.ch; 6 Signal Processing Laboratory (LTS 5), Ecole Polytechnique Fédérale de Lausanne (EPFL) Lausanne, 1015 Lausanne, Switzerland; 7 Department of Neurosurgery, Neurosurgery Clinic, Birgunj 44300, Nepal; trozexa@gmail.com; 8 IRCCS Istituto Delle Scienze Neurologiche di Bologna, UOC Oncologia Sistema Nervoso, 40139 Bologna, Italy; e.franceschi@isnb.it; 9 Functional and Molecular Neuroimaging Unit, IRCCS Istituto Delle Scienze Neurologiche di Bologna, 40123 Bologna, Italy; 10 IRCCS Istituto Delle Scienze Neurologiche di Bologna, 40123 Bologna, Italy

**Keywords:** primary intra-axial neoplasms, connectome, graph-theory, higher-order cognition, quicktome, onco-functional balance

## Abstract

Despite the therapeutical advancements in the surgical treatment of primary intra-axial neoplasms, which determined both a significative improvement in OS and QoL and a reduction in the incidence of surgery-induced major neurological deficits, nowadays patients continue to manifest subtle post-operative neurocognitive impairments, preventing them from a full reintegration back into social life and into the workforce. The birth of connectomics paved the way for a profound reappraisal of the traditional conception of brain architecture, in favour of a model based on large-scale structural and functional interactions of a complex mosaic of cortical areas organized in a fluid network interconnected by subcortical bundles. Thanks to these advancements, neurosurgery is facing a new era of connectome-based resections, in which the core principle is still represented by the achievement of an ideal onco-functional balance, but with a closer eye on whole-brain circuitry, which constitutes the foundations of both major neurological functions, to be intended as motricity; language and visuospatial function; and higher-order cognitive functions such as cognition, conation, emotion and adaptive behaviour. Indeed, the achievement of an ideal balance between the radicality of tumoral resection and the preservation, as far as possible, of the integrity of local and global brain networks stands as a mandatory goal to be fulfilled to allow patients to resume their previous life and to make neurosurgery tailored and gentler to their individual needs.

## 1. Introduction

During the past decade, a growing body of literature demonstrated the significance of the extent of resection (EOR) as a pivotal prognostic factor in both low-grade and high-grade gliomas, documenting its positive impact in the achievement of higher percentages of overall survival (OS) and progression-free survival (PFS) [1]. Indeed, these data confirmed the role of surgery as the first-choice treatment for primary intra-axial neoplasms and introduced the concept of “supramarginal” resection (i.e., beyond tumour boundaries) in both symptomatic and incidental tumours [2]. Traditionally, the perception of supratentorial glioma corresponded to that of a growing mass compressing noble cortical sites, namely those designated to major neurological functions such as movement, language and vision. Consequently, the aggressiveness of the surgical cytoreduction has been conventionally guided by the orthodox subdivision into “eloquent” or “non-eloquent” brain areas in hopes of preserving the fragile equilibrium between the oncological and functional necessities, with few regards to the whole-brain circuitry [3]. This topological philosophy influenced the whole surgical approach to glioma, resulting in acceptable oncological results and in the minimization of intraoperatively induced neurological deficiencies related to the major domains of language, motricity and visual function [4,5]. Notwithstanding these advancements, nowadays patients continue to manifest subtle post-operative higher-order cognition impairments, preventing them from reintegration back into a canonical social life and workforce [6]. Fortunately, past years have witnessed the birth and raising of a completely new scenario in neuroscience, the field of brain connectomics, thanks to the development of the Human Connectome Project (HCP) as of 2010. Indeed, this project paved the way for a profound reappraisal of the traditional concept of a modular brain architecture, starting with the parcellation of each hemisphere into 180 cortical regions, functionally intertwined in harmonious neural networks within a so-called “connectomal” architecture [7]. In contrast to the conventional “localizationalist” dogma, where isolated cortical areas corresponded to specialized functions, brain connectomics favoured the introduction of a meta-networking theory of cerebral functions, in which adaptive behaviour and complex cognition are mediated by a perpetual succession of equilibrium states within and among constantly interacting delocalized neural circuits [8]. Therefore, according to this innovative philosophy, the networks designated for cognition, conation, emotion and behaviour are structurally and functionally constituted by a mosaic of cortical areas organized in a fluid framework densely interconnected by subcortical bundles, in a way capable of achieving the minimal energetic dissipation and the maximal flux of information [9]. Clearly, the actual meaning of this theory lies in the integration of the previous interpretation of brain architecture, founded on the presence of highly specialized and localized modular areas devoted to major neurological functions such as language, motricity and vision, with the idea that higher-order cognitive domains (i.e., cognition, conation, theory of mind, etc.) are instead highly delocalized and sustained by a brain-wide dialogue between sparse intercommunicating networks. Then, thanks to this new concept of brain structural and functional anatomy, neurosurgery is facing a new era of connectome-based tumoral resections, in which the core principle is still represented by the achievement of the ideal onco-functional balance, by maximizing the EOR while avoiding major neurological impairments, but with more attention on global circuitry and more awareness of the risks and benefits of the surgical procedure in a network-wide global perspective. 

The purpose of this review is to provide an overview of the latest advances in the nascent field of brain connectomics applied to neuro-oncological surgery, taking into account graph theory as a key of interpretation of brain networks and focusing on feasible practical applications of connectomics in the pre-operative setting.

## 2. The Connectomal Architecture of Neural Anatomy: Beyond the Concept of “Eloquent” and “Non-Eloquent” Brain

### 2.1. The Development of a Novel Model of Brain Architecture

Since the seminal works of Broca and Wernicke in the definition of the core brain areas responsible for the articulation and perception of language and Jackson’s efforts to identify the primary motor cortex, the foundations of the traditional conception of brain network organization were established and represented as a fixed framework. In this model, major neurological (i.e., motricity, language and visuospatial organization) and higher-order functions (i.e., conation, cognition) were based on highly modular and specialized cortical regions, in which the occurrence of a lesion, either iatrogenic or pathological, would determine irreversible neurological impairments [10,11,12]. The neuroscientific progress and the technological improvements contributed to evolve this dogmatic paradigm, proceeding to make this rather inflexible model more dynamic and fluid. Indeed, as demonstrated by Sawaya et al. in 1998, tumours closer to or inside eloquent areas tend to manifest higher rates of neurological complications, and gross tumour resection (GTR) can still be performed with an acceptable grade of functional impairment, proving that the sole eloquent location did not stand as an absolute contraindication to surgery [13]. This suggests that major neurological functions, traditionally thought to be subserved by highly specialized and isolated brain areas, whose borders are not to be trespassed to avoid major functional impairments, could probably be the product of the dialogue between a more diffuse and sparse ensemble of subnetworks, located in close proximity or even far from the main cortical core (i.e., the frontal operculum—Broca’s area), interconnected by a dense web of subcortical bundles. This rather more diffuse and dynamic model of brain networks ideally justifies the degree of plasticity and compensation that allows for the reorganization of functions that can be seen after surgical interventions in eloquent areas or in the local and global changes in functional and structural connectivity produced by slow-growing lesions such as diffuse low-grade gliomas (DLGGs) or by chronic long-lasting conditions as focal epilepsies, schizophrenia and depression. 

### 2.2. Implications in Epilepsy, Schizophrenia and Depression

Indeed, several conditions such as temporal-lobe epilepsy (TLE) related to mesiotemporal sclerosis and extra-temporal epilepsy (ETLE) due to malformations of cortical development were traditionally interpreted as localization-related diseases, but now have undergone a significative reconceptualization, putting the emphasis on network alterations both at a local and at a global level [14,15]. In fact, network configuration is now recognized to have a preeminent role either in the generation and propagation of drug-resistant seizures or in the development of cognitive and emotional impairments [16]. Apart from theoretical considerations, the connexional signature is being studied and investigated in patients destined to temporal lobectomies and extratemporal lesionectomies because of its potential role in surgical planning and in the estimation of the post-operative prognosis [17,18]. In the case of schizophrenia, several studies proved that functional and structural connexional alterations have a central role in the genesis and natural history of the disease and in the definition of its clinical and cognitive subtypes, even if further evidence has to be documented to remodulate the clinical management of this condition [19]. Regarding depression, Yun and Kim recently published a review that collects the most updated neuroradiological study findings concerning the organizational signature of structural and functional networks in major depressive disorder (MDD), highlighting among other findings, using graph-theoretical metrics, a lowered degree of functional integration between the default mode network (DMN) and limbic networks and decreased structural connections of medial temporal–parietal and dorsolateral prefrontal cortices with other brain regions in patients with MDD versus HC, supporting the idea of MDD as a network disease and reinforcing the reinterpretation of brain architecture in a more dynamic and fluid mosaic of interacting networks [20]. 

### 2.3. Implications in Neuro-Oncology

In neuro-oncology, as suggested by Duffau et al., the traditional conception of a modular brain organization, characterised by a fairly rigid subdivision of the brain into eloquent and non-eloquent areas, implied at least two major negative consequences in the surgical approach to slow-growing lesions such as DLGG [3]. Specifically, if the tumour was situated in the presumed “eloquent” cortical regions, patients were less likely to be admitted to surgery, thereby causing a significative loss from an oncological point of view. On the contrary, if the tumour involved areas deemed “non-eloquent”, patients were more likely to be treated without the aid of brain mapping techniques, augmenting the risks of functional impairments. 

However, as demonstrated by a sample study from the database of the Glioma Outcome Project published in 2005, the lateralization of the neoplasm, and therefore the involvement of either the dominant hemisphere (DH) or the non-dominant hemisphere (nDH), does not stand as an independent prognostic factor for the prediction of functional outcomes, except for language [21]. Consequently, hemispheric dominance, for a long time closely related to the concept of eloquence, does not fully explain the complexity of neural architecture and the heterogeneity of neurocognitive deficiencies occurring both prior to and after the surgical treatment. With these premises agreed upon, the ground-breaking information gathered through the completion of the HCP paved the way for a complete reappraisal of the structural and functional organization of neural connectivity, offering an innovative and broader understanding of the wiring diagram of the brain. Particularly, a great amount of the current knowledge on neural architecture has been derived from graph-theoretical analyses of resting-state functional MRI (rsfMRI), which allowed for looking at neural circuitry from a fairly mathematical perspective, thereby representing cortical areas and their connections as a set of nodes interconnected by edges and demonstrating that the intrinsic cerebral connectivity is organized as a small-world network with significant modularity and populated by highly connected hub-regions [22]. This network architecture can assume an infinite number of synchronization patterns by relying on an incessant succession of equilibrium states through a constant communication between functional systems, resulting in an adaptative behaviour tailored to environmental demands as well as in a pronounced neuroplastic potential responsive to physiological and pathological stimuli [8]. Aside from more technical graph-theoretical observations, the information gathered from these analyses allowed the description of seven major networks participating in higher-order cognition, of which three constitute the so-called “main cognitive networks”: the default mode network (DMN), the central executive network (CEN) and the salience network (SN) [23]. Predictably, recent evidence showed that the disruption or the abnormal connectivity in these major systems determines the impairments of higher-order functions and relates to neurocognitive decline and could also form the foundation of several psychiatric disorders such as schizophrenia, depression and anxiety [24]. Therefore, the HCP and its corollary studies and analyses have provided a plethora of innovative information to further understand the complex nature behind human cognition and adaptive behaviour. Particularly, a connectomic atlas of the human brain describing the functional and structural connectivity of all 180 cortical parcellations has recently been published, according to the HCP authors [25]. However, the fact of owning this vast amount of information does not necessarily mean that the following passage of drawing clinically actionable inferences is automatically smooth and straightforward. Indeed, the current general knowledge about a neural network suggests that lesioning the cores of the aforementioned main cognitive networks, regarding both the individual parcellations essential to the network itself and the interconnections between those critical parcellations, provokes severe cognitive impairments. On the other hand, other fundamental concepts remain currently undefined, such as the precise quota of hubs and fibres that should be preserved and not disconnected to avoid a compromission of the network and the subsequent neurocognitive sequelae [26]. Therefore, despite the improved knowledge in the understanding of the wiring diagram of the human brain and the advancements made in the conservation of noble neurological domains, the field of connectomics applied to neuro-oncological surgery is still nascent and requires further steps ahead to achieve a gentler and safer neurosurgical act.

In conclusion, connectomics provides a reinterpretation of the concept of eloquence compared to the traditional topological philosophy. Indeed, rather than merely considering the tumour as an expansive process localized in a certain cortical area, but as a disturbing element plunged in a densely intercommunicating ensemble of networks, with which a mutual dialogue occurs (causing the actuation of both locoregional and global mechanisms of functional reallocation), it is possible to reinterpret the indications and the feasibility of a given surgical strategy and to predict the probability of post-operative neurological deficits from a perspective that takes into account a brain-wide web of neural connections among adjacent or distant brain areas, rather than from that of a rigid subdivision into “eloquent” or “non-eloquent” territories.

## 3. Application in Cancer Neuroscience: Connectomics for a Gentler and Safer Neurosurgical Act

### 3.1. Connectome-Based Brain Mapping and Graph-Theoretical Analysis

**Figure 1:**
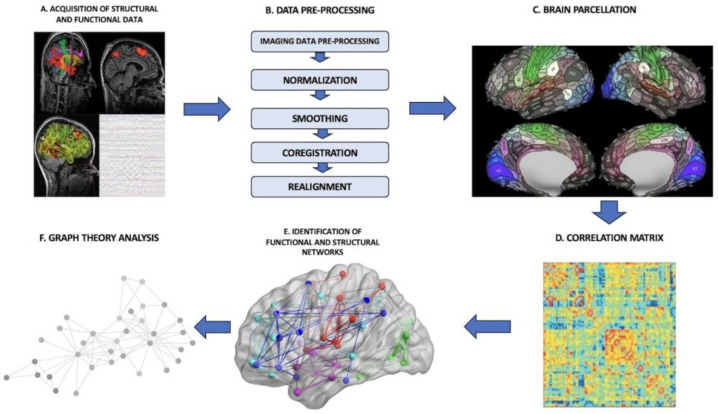
**General connectome-analysis pipeline**. (**A**) Acquisition of structural or functional data; (**B**) data pre-processing phase; (**C**) brain parcellation phase; (**D**) construction of a correlation matrix to establish the connectedness among the various parcels; (**E**) identification of functional brain networks; (**F**) application of graph-theoretical analyses to define the properties of networks.

In the pre-operative setting, one of the possible applications of a connectome analysis is to derive an individualized connectomic map for each patient from rsfMRI sequences. The process requires us to follow a specific pipeline constituted by several phases (Figure 1): (I) There is the acquisition of the imaging data, that is, functional data from rsfMRI or MEG, to construct a functional connectome and, vice versa, DTI or anatomical MPRAGE sequences to build a structural connectome. Depending on the source used to construct the connectome, either functional or structural, the final provided information is different. In the functional connectome, brain areas are paired by defining metabolic or electric connections. In the structural connectome, the final result reflects physical connections among brain areas made by subcortical tracts and white matter bundles. (II) The brain is split into distinct regions through a process known as parcellation, where each parcel represents a so-called “node”. (III) In the case of functional connectomes, the connections among the several nodes are established by extracting the blood-oxygen-level-dependent (BOLD) time series from each parcel and compared with the time series from all other parcels. (IV) The connectedness among the various cortical areas is eventually displayed as a correlation matrix. (V) Further analyses are processed through the application of graph-theoretical methodologies to investigate the properties of individual regions, the links between these regions or the effect of lesioning or perturbation of specific networks [27].

Graph theory is a branch of mathematics that studies networks of connected elements and it is frequently applied in the analysis of complex systems. A graph is defined by point-like components, nodes or vertices, and by the connections between them, edges or vertices. Therefore, neural networks can be represented as a set of nodes (single neurons, neuronal populations, macroscopic brain areas) and edges (structural and functional interactions between them) [28,29]. 

Particularly, several graph-theoretical measures assume a significative clinical relevance in the setting of patients affected by a brain tumoral disease (Table 1). These include

- **Node degree**: the number of connections of each parcel.
- **Clustering coefficient**: a measure of network segregation or local specialization and it quantifies the number of connections that exist between the nearest neighbours of a node as a proportion of the maximum number of possible connections.
- **Path length**: a measure of network integration or global information flow and it represents the minimum number of edges (i.e., connections) that must be traversed to go from one node to another.
- **Efficiency**: the reciprocal of path length and it can be local or global. For higher values of path length, there is a reduction in the efficiency of the information flux.
- **Hubs**: nodes with high degree or high centrality.
- **Centrality**: it describes the importance of a node in the context of the overall network by quantifying the number of shortest paths between all the other node pairs in the network that pass through the given node.
- **Robustness**: it reflects the changes in structural and functional integrity of a network after the removal of individual node(s) or edge(s).
- **Information centrality**: it represents the percentage change in global efficiency due to the removal of a single node.

The last two aforementioned properties can be virtually assessed by performing the so-called random error and targeted attack. Namely

- **Random error**: removal of casually selected nodes with subsequent measurement of the changes in network properties.
- **Targeted attack:** suppression of specific nodes based on individual features such as their degree, centrality or clustering [30,31].

**Figure 2:**
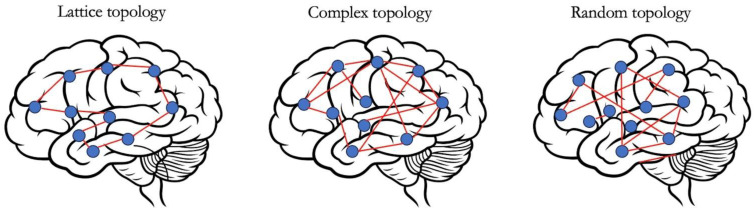
**Models of network topology**. (**Left panel**) A lattice network is characterised by an array of nodes solely connected with their adjacent neighbours. Hence, it presents a high degree of segregation combined with a low wiring cost (due to the absence of high wiring cost long-range connections). However, the local and global efficiency is low (i.e., the efficiency of the information flow across the entire network), since travelling from one end of the network to the other requires traversing a large set of nodes. (**Right panel**) A random network is the opposite of a lattice network. Indeed, it is characterised by random connections among nodes and it combines a high wiring cost with efficient integration, despite the low segregation. (**Middle panel**) Neither lattice nor random models represent accurate models of complex networks. The human brain seems to stand between these two extremes. Indeed, there are clusters of lattice-like short-distance connections between spatially neighbouring nodes (organized as modules) to minimize wiring cost. Brain networks also include high-cost connections (i.e., long-distance short-cuts between connector hubs in different modules and different anatomical regions) to increase the efficiency of information processing and constitute the substrata for adaptive behaviour.

The evidence collected using a graph-theoretical analysis suggests that a fundamental property of brain networks is that they demonstrate **small-world features**. This property allows the simultaneous fulfilment of the needs of segregation (i.e., localized specialization, which correlates with a high clustering coefficient) and integration (i.e., an efficient local and global information flow, correlated with a high value of global efficiency and reciprocally short path lengths) that are critical requirements for a fluid exchange If information across neural circuits, resulting in adaptive behaviour (Figure 2). **Modularity** is another property of complex networks and it is assessed by grouping together strictly interconnected nodes to identify modules and submodules according to hierarchical clustering algorithms [32]. Each module, which therefore represents the elementary unit of a complex network, is populated by densely interconnected nodes that constitute the so-called hubs, either **provincial hubs** if connected mainly to elements in their own module or **connector hubs** if related to nodes in other modules [33]. Hence, modularity reflects local specialization, but contextually allows cost-effective integration by establishing sparse long-range connections between modules [28]. 

Hence, the rich lexicon that characterises graph-theoretical analyses offers a new way of describing structural and functional cores in terms of hubs, modules, efficiency and other measures, which can be the foundation of an innovative approach for the exploration of brain activity [28]. 

As already mentioned, in the field of neuro-oncology, the primary goal is to maximize the EOR while preserving an intact neurocognitive status. Alongside other effective brain mapping methodologies such as intraoperative cortical stimulation, which allowed significative advancements in the achievement of a better onco-functional balance, graph-theoretical analyses of the individual connectome could provide essential information regarding the reactive adaptative processes mediated by the brain in response to the presence of focal lesions (i.e., both tumoral and surgically induced ones) and even predict dynamic changes in network architecture, thereby revealing their plastic potential possibly in a reparative manner [28]. Indeed, an abnormal configuration of brain connectivity has been detected in patients affected by brain tumours and could explain the battery of common cognitive impairments found in this population. In fact, most patients suffer from several cognitive deficits, concerning attention, working memory, psychomotor speed and other executive functions, that can hardly be explained by the effect of a focal growing mass [34]. Indeed, in these cases, brain network architecture has been found to present less small-world features in favour of a more random and less organized configuration, globally and not just locally. Specifically, a valid indicator for cognitive processing is represented by global efficiency, which in patients affected by DLGG is impaired and seems to be responsible for their intellectual decline [35]. The clinical relevance of graph-theoretical analyses materialises in the definition of the correlation between network parameter alteration and clinical symptoms of neurocognitive impairment. Contextually, the degree of corruption of network features has been correlated with functional deficits regarding localized and diffuse cortical areas impaired by a reduction in global efficiency and small-worldness, as well as with lower IQ scores [35]. Lastly, the effects of surgery on network organisation have been measured post-operatively, showing the return to a more organised and less random architecture compared to the more disrupted pre-operative configuration [36]. Additionally, the seizure outcome has also been influenced by the post-operative conversion to an ameliorated network configuration [37].

From a pre-operative perspective, graph theory could integrate with intra-operative brain mapping methodologies, such as direct electrical stimulation (DES), whose main constraint is represented by being capable of mapping only single networks and local functions. Conversely, a connectome analysis balances the traditional localizationalist philosophy and the theory of meta-network connectivity in a small-world framework model, hence allowing for a more profound understanding of the effects of focal lesions (both tumoral and surgically induced) on the whole neural ecosystem, observing from a wider angle the known global alterations in connectivity that occur at intra-lobar, intra-hemispheric and inter-hemispheric scales [31]. Indeed, a graph analysis allows an accurate prediction of inducible cognitive deficits by assessing the vulnerability of a specific network, which could depend either on its susceptibility to targeted attacks or on its neuroplastic potential. Practically, brain regions containing networks designated to critical functions or with a particularly low plastic potential will be treated in a safer way, thus avoiding extensive resections. On the contrary, regions with limited functional relevance or provided with a more flexible potential for reorganisation could be more aggressively treated with little or absent functional consequences [38,39]. 

In conclusion, the application of connectomics and a graph analysis in neurosurgery is undoubtedly a nascent field and several improvements are required to allow a routinary use of these methodologies and make them applicable to standard clinical MRI scans. Notwithstanding these observations, the revolutionary perspective of considering the brain as the product of its connections, rather than a mosaic of isolated functional areas, offers the alluring opportunity to introduce innovative tools for the production of realistic models of brain lesions to use pre-operatively, which can contribute jointly with intraoperative techniques to the surgical treatment of primary intra-axial neoplasms to enhance the onco-functional balance.

### 3.2. An Insight into the Application of Quicktome^TM^ for the Identification of Brain Networks in Patients Affected by Primary Intra-Axial Neoplasms

As already mentioned, the main goal of neuro-oncological surgery is to enhance the EOR while providing the best possible neurological outcome. However, the equilibrium between the effectiveness of the surgical act and the occurrence of QoL-impairing post-operative neurological deficits is fragile. Moreover, if we consider that QoL can be severely affected by subtle deficiencies regarding higher-order neurological domains such as personality, memory, executive and semantic functions or metacognition, it is evident that a deeper knowledge of the “non-traditional” eloquent areas is needed to provide an all-around evaluation of every single-case to ensure the best treatment to the individual patient. Then, the traditional concept of a modular subdivision of the brain into eloquent or non-eloquent areas seems to not fully encompass its complexity and wide-scale interactions anymore. On the other hand, the integration of this traditional philosophy with the innovative perspective provided by the discipline of connectomics could allow the definition of a brain mapping paradigm in which the outcome, that is, a given neurological function, is the product of a choral dialogue among both adjacent and distant networks and subnetworks, instead that of highly modular and isolated noble cortical sites. 

Several imaging techniques are routinely used in the pre-operative neurosurgical setting to identify the relationship between the tumour and eloquent cortical sites. fMRI, DTI and repetitive navigated transcranial magnetic stimulation (rTMS) are effective methodologies that, apart from intrinsic technical limitations, require expert personnel, and are time-consuming, expensive and often not available at many institutions. Moreover, a crucial limitation is represented by the fact that many software options devoted to the identification of networks and relevant functional areas rely on the normalization of the patient’s brain images to standardized normal brain atlases, thus without regards to the alterations in shape and size determined by the presence of an expansive oedematous mass.

Quicktome^TM^ (Ominiscient Neurotechnology; Sydney, Australia) is a Food and Drug Administration (FDA)-approved novel software based on the HCP that uses machine learning and reparcellation techniques to accurately identify the brain network and that can be integrated into conventional neuronavigation systems. The main advantage of this cloud-based software is that it provides an individual version of the HCP Multi-Model parcellation version 1 atlas (HCP-MMP1 atlas) [7] based on DTI-structural connectivity and not on anatomy-based methods to overcome the limitations induced by a distorted neuroanatomy [40]. This system was created firstly by training a machine learning model on 200 normal adult brains according to a specific processing pipeline of DTI and T1 sequences [40]. Secondly, the HCP-MMP1 atlas in Montreal Neurologic Institute (MNI) space was warped onto each brain to establish structural connectivity between each pair of cortical parcels, eight subcortical regions per hemisphere and the brainstem. This step generates both a structural connectivity-based adjacency matrix with its feature vectors for each voxel and a centroid of the parcellation used to enclose the voxels studied for the assignment to a given parcellation in a plausible area close to its typical position. Therefore, the feature vectors and the centroid of parcellation are used to determine whether each specific voxel pertains to a given parcel or not and if so to assign the voxel to that parcel according to structural connectivity and not merely the anatomic-based warping of a connectomic atlas to a single-subject brain [40,41]. 

**Figure 3:**
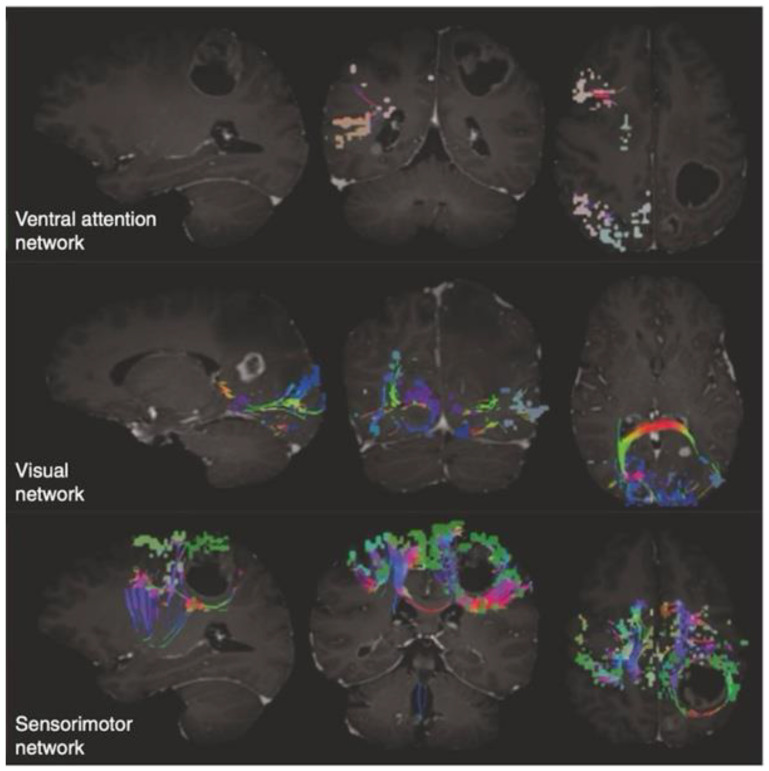
Quicktome^TM^ (Omniscient Technology; Sydney, Australia) network rendering in the setting of primary intra-axial tumours. A representation of different network–tumour interaction scenarios. (**Above**) Network not compromised/not at risk: the tumour and the perilesional oedema do not involve the network under examination, which in this case is the ventral attention network; (**Middle panel**) network at risk: either the tumour or the perilesional oedema causes a displacement in the network or the position of the network is susceptible to damage due to its proximity to the mass or to the surgical trajectory; (**Below**) network compromised: the network shows damaged or missing tracts/parcellations. Tracts are shown by multicolour lines, parcellations by group of dots of the same colour. Morell AA, Eichberg DG, Shah AH, Luther E, Lu VM, Kader M, Higgins DMO, Merenzon M, Patel NV, Komotar RJ, Ivan ME. Using machine learning to evaluate large-scale brain networks in patients with brain tumors: Traditional and non-traditional eloquent areas [42].

Eventually, the rendering of the individual brain networks, tracts and parcellations can be visualized on demand through an Internet browser and easily exported to the institution PACS system (Figure 3). 

Several studies explored the application of Quicktome to the routinary neurosurgical workflow in the setting of primary intra-axial tumours. First, the initial question to answer had to regard the feasibility of its integration in standard frameless stereotactic systems and the amount of time taken to elaborate the raw imaging data and translate them into meaningful brain maps in a reasonable amount of time. Recent studies have retrospectively collected cohorts of patients that have underwent a craniotomy for primary intra-axial tumours and have demonstrated the successful marriage of the neuronavigation platform with the brain maps produced by Quicktome in the totality of the cases [40,41,42]. Contextually, a study from Morell et al. underlined that the processing pipeline is fully automated in its pre-processing and processing steps with an average elaboration time of 52 min (SD: 10.82, range: 17–81), thus excluding that the integration of this platform in the surgical workflow could result in a time-consuming procedure [42].

Second, the other question that has to be answered is whether this system can provide meaningful information that can influence the surgical decision-making process and the pre-surgical planning, and whether it can help in the prediction or explanation of post-operative neurological deficits, either belonging to the basic function realm (language, motricity, vision) or to the so-called “non-traditional” eloquent domains (DMN, CEN, SN, etc.) underpinning higher-order cognition. The aforementioned studies were designed to retrospectively collect a population of patients who underwent a craniotomy for a primary intra-axial tumour and to evaluate whether the post hoc application of Quicktome could provide adjunctive information. As expected, it emerged that patients presenting focal neurological deficits showed a significantly higher number of altered networks compared to patients without signs and symptoms (3.42 vs. 2.19 networks, *p* < 0.001). However, less predictably, patients without neurological deficits still manifest a significant quota of disrupted networks, most of them pertaining to non-traditional eloquent areas [42]. Precisely, in the study of Morell et al., non-traditional eloquent networks were at risk of involvement or involved in 93% of the cases [42]. In a recent study regarding the involvement of non-traditional eloquent cortical sites in insulo-sylvian gliomas, Wu et al. documented a 98% disruption of networks such as the DMN, CEN, SN and other relevant large-scale circuits [40]. Therefore, it is reasonable to hypothesize that given the high chance of alteration of the networks subserving higher-order cognition, in most cases the consequential deficits are misdiagnosed and a correct pre-operative assessment with adequate neuropsychological batteries of tests should be required. At the same time, the opportunity to visualize these networks and their proximity or involvement with the tumoral mass could come in aid in the surgical planning phase. In fact, owning a structural connectivity-based brain map that is not influenced by the effects on brain shape and size determined by a growing lesion could help in the definition of a more informed surgical trajectory rather than one based essentially on raw neuroradiological data. Moreover, it is worthwhile to consider that many white matter bundles are composed of fibres of variable relevance, not all of which are essential to cognitive function, for example, the superior longitudinal fasciculus (SF) or the arcuate fasciculus (AF). According to a connectome-based approach, the surgical act could be tailored to sacrifice those fibres necessary to grant an adequate EOR, while preserving those crucial to maintain an acceptable cognitive function [40]. 

Clearly, there are some drawbacks to the use of such a nascent technology like Quicktome. First, the main studies are based on retrospective cohorts of patients and thus need further prospective and systematic validations. Second, same as many other adjuncts to conventional neuronavigation systems, it does not consider the brain shift during tumoral excision. Third, given that the identification of complex networks subserving higher-order function could potentially influence the surgical act, it is of utmost importance to standardize the implementation of more accurate neuropsychological batteries of tests, which are not routinely applied and should be the object of future investigations, to be able to recognize the potential neurological impairments that could derive from trespassing tumour borders during the excision.

Apart from the obvious limitations of a nascent technology, which imply the need of further studies and external generalized validations, the perspective offered by these new methodologies is alluring. The potential benefits derived from the reinterpretation of brain architecture could help in the process of refinement of the surgical planning phase to make the act gentler and more tailored to the single-subject distorted neuroanatomy and network configuration. 

## 4. Conclusions

The ground-breaking innovations introduced by the discipline of connectomics paved the way for a better understanding of the complex functional and structural architecture of the brain. The interpretation of the substrata underpinning higher-order cognition and complex behaviour has switched from the traditional localizationalist dogma to a new meta-networking theory of brain functions. The analysis of brain connectivity through graph-theoretical measures could improve the understanding and interpretation of the neurological substrata of complex cognition and adaptative behaviour. The application of a connectome-based approach through the implementation of novel technologies based on machine learning offers new perspectives in the setting of intracerebral surgery for primary intra-axial neoplasms. 

## Figures and Tables

**Table 1:** Glossary of graph-theoretical measures.

Measure	Definition
Node degree	The number of connections/edges of a specific node
Clustering coefficient	The number of connections that exist between the nearest neighbours of a node as a proportion of the maximum number of possible connections
Path length	It is a measure of network integration or global information flow and represents the minimum number of edges (i.e., connections) that must be traversed to go from one node to another
Efficiency	It is the reciprocal of path length and can be local or global
Hubs	Nodes with high degree or high centrality
Centrality	It quantifies the number of shortest paths between all the other node pairs in the network that pass through a specific node. Therefore, it is a measure that describes the importance of that specific node in the overall network
Robustness	It reflects the changes in structural and functional integrity of a network after the removal of individual node(s) or edge(s)
Information centrality	It is a measure that describes the percentage change in global efficiency due to the removal of a single node
Random error	It consists in removing casually selected nodes and then measuring the change in network properties
Targeted attack	It consists in the suppression of specific nodes based on individual features such as their degree, centrality or clustering
